# Association between type 1 diabetes and neurodevelopmental disorders in children and adolescents: A systematic review and meta-analysis

**DOI:** 10.3389/fpsyt.2022.982696

**Published:** 2022-11-22

**Authors:** Xue-Ni Xie, Xue Lei, Chun-Ye Xiao, Ya-Min Li, Xian-Yang Lei

**Affiliations:** ^1^Clinical Nursing Teaching and Research Section, The Second Xiangya Hospital, Central South University, Changsha, China; ^2^School of Public Health, Kunming Medical University, Kunming, China; ^3^School of Psychology, The University of Queensland, St Lucia, QLD, Australia; ^4^School of Nursing, Jinan University, Guangzhou, China; ^5^Office of the President, Central South University, Changsha, China

**Keywords:** type 1 diabetes mellitus (T1DM), neurodevelopmental disorders (NDDs), prevalence, autism spectrum disorder (ASD), meta-analysis, attention deficit hyperactivity disorder (ADHD)

## Abstract

Type 1 diabetes and neurodevelopmental disorders are common chronic conditions in childhood and adolescence, and having one may lead to an increased chance of developing the other. Type 1 diabetes mellitus is mainly manifested by elevated blood glucose, while neurodevelopmental diseases are composed of a variety of diseases, which are relatively complex. The purpose of this meta-analysis was to find out the prevalence of type 1 diabetes-related neurodevelopmental disorders in children and adolescents and to explore the potential association between neurodevelopmental disorders and type 1 diabetes. PubMed, Embase and Web of science databases were searched from the inception to May 22, 2022 to identify relevant studies, Finally, 24 original studies were included in the meta-analysis. Prevalence estimates for neurodevelopmental disorders in the type 1 diabetes adolescent and their 95% confidence intervals were pooled using random effects models. The pooled estimates for autism spectrum disorders (ASD) and attention deficit hyperactivity disorder (ADHD) in the type 1 diabetes population were 1.2 and 5.3%, respectively, both of which are higher than the 2019 global prevalence of ASD and ADHD in the general population. The results of the subgroup analysis showed that the prevalence of ASD and ADHD in the T1DM population tended to increase with age. In conclusion, there may be a potential link between the occurrence of type 1 diabetes mellitus and the development of neurodevelopmental disorders in children and adolescents, but more relevant studies are needed to understand the link between the underlying pathogenesis of type 1 diabetes and neurodevelopmental disorders.

**Systematic review registration:**

[https://www.crd.york.ac.uk/PROSPERO/], identifier [CDR42022333443].

## Introduction

Neurodevelopmental disorders (NDDs) are a group of complex disorders that are not easily conceptualized. In the DSM-5, NDDs are defined as a group of disorders that occur during development, including autism spectrum disorders (ASD), attention deficit hyperactivity disorder (ADHD), intellectual disability (ID), communication disorders (CD), specific learning disabilities (SLD), and motor disorders (MDs) (1, 2). They are characterized by childhood onset with delays in developmental domains such as cognition, executive functioning, language/communication, social functioning, and adaptive behavior (3), besides, people with ASD also have social and interaction difficulties (4). There are gender differences in the onset of NDDs. The diagnosis rate of males is higher than that of females, and the onset risk of males is 2–4 times higher than that of females (5–8). Regarding to the most common NDDs, previous studies published specific prevalence: 1/6 (17.4%) of American children aged 2–8 suffer from mental, behavioral or developmental disorders (9). In 2018, the global average prevalence of ADHD in school-age children was 5% (range: 2–7%) (10). In addition, according to the Centers for Disease Control, the prevalence of ASD among 8-year-olds in the United States increased from 0.67% in 2000 and 2002 to 2.3% in 2018 (11–13). The additional health, social care and education costs associated with childhood mental illness were estimated at £1.47 billion in 2008 in Britain, which impose a significant social and economic burden (14–16). Furthermore, mental disorders are listed as one of the top 10 causes of the global burden (17) and are seen as a major contributor to the burden of disease (18). Therefore, it is suggested to investigate causal pathways between mental disorders and other severe health issues.

Diabetes is another chronic disease that can be extremely dangerous, which can seriously damage many systems of the body, especially nerves and blood vessels. It is defined as a group of metabolic diseases characterized by hyperglycemia caused by defective insulin secretion, defective insulin action or both. There are two types of diabetes, type 1 diabetes mellitus (T1DM) and type 2 diabetes (19). The prevalence of diabetes is increasing year by year, with 451 million adults worldwide living with diabetes as of 2017, and the number is expected to rise to 693 million by 2045 (20).

Type 1 diabetes is characterized by elevated blood glucose levels due to insulin deficiency caused by loss of pancreatic b-cells, and it is a common chronic disease in children and adolescents, along with mental disorders (2, 21, 22). Studies have shown that T1DM brings great psychological burden to children and adolescents, and greatly increases the risk of mental health problems (23–25). NDDs are more common in children with T1DM compared to the general population (26–28). Vice versa, NDDs may also affect blood glucose control and increase the risk of diabetes complications (29). The International Society for Pediatric and Adolescent Diabetes (ISPAD) notes that mental health problems can lead to serious impacts and long-term consequences in young people with T1DM (30). However, no comprehensive study has examined the relationship between NDDs and T1DM in children and adolescents.

NDDs and T1DM are common in children and adolescents, may have similar pathogenesis, and may occur together. The purpose of this study was to investigate the prevalence of T1DM and NDDs in children and adolescents through meta-analysis and to explore the potential association between these two diseases.

## Materials and methods

The Preferred Reporting Items for Systematic reviews and Meta-Analyses (PRISMA) guideline were followed by this systematic review (31). The protocol has been registered in PROSPERO (CDR42022333443).

### Search strategy

We searched Embase, PubMed, and Web of Science databases for relevant articles published as of May 22, 2022. We selected subject terms and keywords related to diabetes, autism, ADHD, ID, CD, SLD, and motor disabilities in different databases, and the detailed retrieval strategy is shown in Supplementary Table 1. Additional studies were identified by manually reviewing of the references of included studies. The study selection process was completed independently by two researchers (X.L. and XN.-X.).

### Study inclusion criteria

The inclusion criteria of the studies were as follows: (a) the age of population was 18 years old or below; (b) the population is accompanied by T1DM with NDDs or NDDs with T1DM; (c) The reported study must be published in English; (d) Prevalence estimates or raw data can be used to calculate the prevalence are available in the study; (e) The study to be included must be original studies, and reviews or systematic reviews are excluded. All papers that meet the criteria will be included in the systematic review.

### Data extraction and quality assessment

A standardized form was used to extract the following data from the included studies: Author and year of publication, study content (country, study setting, data source, study period, sample size, age, percentage of males), diagnosis and criteria of T1DM and NDDs, outcome (the prevalence of NDDs in T1DM or the prevalence of T1DM in NDDs). If the prevalence estimates were not directly provided in the study, raw data from the article are collected and calculated to obtain prevalence. In addition, we also extracted the types of scales used in the diagnosis of different diseases and the judgment criteria used in the table, which are respectively, expressed as diagnosis and criteria in the table header (Supplementary Table 2). Two researchers (X.L. and XN.-X.) extracted data independently, and disagreements, if any, were resolved through discussion with the third author (YM.-L).

The 14-item rating scale National Institutes of Health Study Quality Assessment Tool for Observational Cohort and Cross-sectional Studies were used to assess the quality of each included study. The quality assessment was performed independently by two researchers (X.L. and XN.-X.) and resolved by negotiation or discussion with the third author (XY.-L).

### Statistical analysis

The main outcome of this meta-analysis summarizes the prevalence of NDDs in individuals with type 1 diabetes. Data from people with T1DM with NDDs were analyzed for comparison. Prevalence estimates in each study were weighted by sample size and number of cases and then pooled through meta-analysis. *I*^2^ statistics were used to evaluate heterogeneity between studies (32). *I*^2^-value less than 30% was considered as low heterogeneity, greater than 60% was interpreted as substantial heterogeneity, and between 30 and 60% was considered as moderate heterogeneity. Publication bias was assessed by visually observing the symmetry of funnel plots and quantitative Egger (33) and Begg (34) tests. If publication bias is found, Duval Tweedie’s “trim and fill” method is used to obtain adjusted estimates (fill estimates) (35). We also looked at the study setting (population-based, clinically based), geographic region (North America, Asia, Europe, Africa), survey period (≤2010, >2010), age range (2–12 years old, ≥ 13 years old) and diagnosis/criteria (single standard, both standard). All statistical analyses were performed using Stata 17.0 software.

## Results

### Study selection and characteristics

**FIGURE 1:**
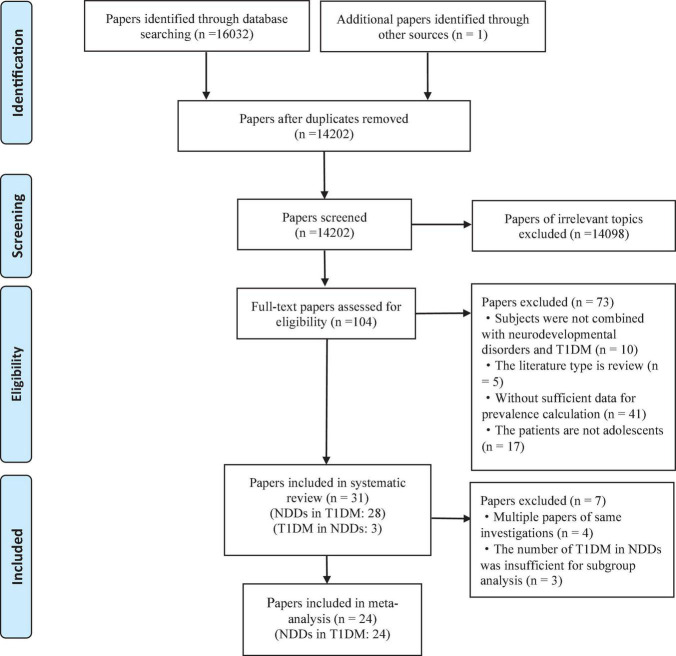
PRISMA flow chart.

The PRISMA diagram for studying the selection process is shown in Figure 1. Among the retrieved studies, a total of 31 met the inclusion criteria (28 were related to NDDs in T1DM, and 3 were related to T1DM in NDDs) and were included in the systematic review. In the included studies, we excluded studies from the same investigation sources and studies related to T1DM in NDDs that were too small to be meta-analyzed. Finally, 24 studies related to NDDs in T1DM were included in the meta-analysis.

All of the studies were conducted between 1973 and 2019, with 20 population-based studies and 11 clinical-based studies (Supplementary Tables 2, 3). As part of the included papers were conference papers, the total number of papers participating in the quality evaluation was 21. The NIHSQAT (National Institutes of Health Research Quality Assessment Tool) was used to score the quality of all articles included in the analysis with full text, with all articles between 6 and 10 points in quality. A total of four items (20.0%) were rated as excellent (≥10 points) and 16 items (80.0%) were rated as fair (≥6 points). The results of the quality assessment are presented in detail in Supplementary Tables 4, 5.

### Autism spectrum disorders/deficit hyperactivity disorder in children and adolescents with type 1 diabetes

Studies on the T1DM and ASD sample were published between 1973 and 2017 and included three clinically based studies and eight population-based studies (22, 23, 26, 36–44). Four studies were conducted in North America, six in Europe. Studies on a sample of patients with T1DM and ADHD, both published between 1973 and 2019, included 14 population-based studies and seven clinically based studies (19, 20, 23, 26, 40, 41, 45–60). One study was conducted in North America, 11 in Europe, one in Africa, and eight in Asia. Of the ASD sample studies, one study (14.3%) was rated as good quality (score ≥ 10), and six studies (85.7%) were rated as general quality (score ≥ 6). Of the ADHD sample studies, two studies (14.3%) were rated as good quality, and 12 studies (85.7%) were rated as general quality.

The prevalence of ASD and ADHD in children and adolescents with T1DM was 1.2% (95%CI: 0.9–1.6%) and 5.3% (95%CI: 4.3–6.4%), respectively (Supplementary Figures 1, 2). Subgroup analysis showed that the prevalence of ASD among adolescents/children with T1DM was 1.1% in population-based studies, 6.1% in clinical studies, 1.0 and 1.3% in Europe and North America, 0.9 and 1.4% before and 2010 and after 2010, respectively. The prevalence was 0.8% in 2–12 years and 1.4% in 13–18 years. The prevalence of ADHD was 4.8% in population studies and 7.7% in clinical studies, and 4.0% in Europe, 28.4% in North America, 17.9% in Asia and 33.3% in Africa. The prevalence in 2010 and before and after 2010 was 2.0 and 6.7%, respectively. The prevalence was 2.7% in 2–12 years and 6.7% in 13–18 years, respectively (Tables 1, 2).

**TABLE 1:** ASD in T1DM populations using random-effects meta-analysis and subgroup meta-analysis.

Variables	No. of studies	Prevalence (95%CI)	*I*^2^ %	*p*_*heterogeneity*_	Egger test	Begg test	No. of missing studies	Filled prevalence (95%CI)

Analysis for ASD	10	1.2 (0.7, 1.6)	*96.7*	<0.001	0.043	0.283	0	NA
Study setting								
Population-based	8	1.1 (0.7, 1.5)	97.3	<0.001	0.067	0.138	0	NA
Clinic-based	2	6.1 (4.8, 17.1)	93.2	<0.001	NA	0.317	NA	NA
Geographical area								
North America	4	1.3 (0.9, 1.6)	68.8	0.022	0.018	0.174	0	NA
Europe	6	1.0 (0.6, 1.5)	96.7	<0.001	0.117	0.573	1	1.0 (0.5, 1.5)
Study period								
≤2010	4	0.9 (0.6, 1.2)	78.8	0.003	0.294	0.174	0	NA
>2010	2	1.4 (0.9, 1.9)	79.2	0.028	NA	0.317	0	NA
Age								
2–12	5	0.8 (0.3, 1.3)	96.7	<0.001	0.322	1.000	1	0.7 (0.2, 1.3)
13–18	2	1.4 (0.9, 1.9)	79.2	0.028	NA	0.317	0	NA
ASD diagnosis/criteria								
Standard diagnosis	3	1.4 (1.0, 1.7)	68.3	0.042	0.171	0.117	0	NA
Standard criteria	5	1.1 (0.7, 1.5)	94.4	<0.001	0.255	0.624	0	NA
Standard diagnosis/criteria	2	1.5 (1.2, 1.8)	62.4	0.103	NA	1.000	0	NA
T1DM diagnosis/criteria								
Standard diagnosis	3	1.4 (1.0, 1.7)	68.3	0.042	0.171	0.117	0	NA
Standard criteria	2	1.0 (0.4, 1.6)	92.8	<0.001	NA	0.317	1	0.7 (0.2, 1.2)
Standard diagnosis/criteria	1	1.3 (1.0, 1.6)	NA	NA	NA	NA	0	NA

**TABLE 2:** ADHD in T1DM populations using random-effects meta-analysis and subgroup meta-analysis.

Variables	No. of studies	Prevalence (95%CI)	*I*^2^ %	*p*_*heterogeneity*_	Egger test	Begg test	No. of missing studies	Filled prevalence (95%CI)

Analysis for ADHD	18	5.3 (4.3, 6.4)	*96.7*	<0.001	0.002	0.197	7	3.8 (2.7, 4.8)
Study setting								
Population-based	11	4.8 (3.5, 6.0)	97.0	<0.001	0.039	0.184	4	3.5 (2.2, 4.8)
Clinic-based	7	7.7 (5.4, 10.0)	94.3	<0.001	0.009	0.099	1	7.0 (4.6, 9.4)
Geographical Area								
North America	1	28.4 (17.4, 39.4)	NA	NA	NA	NA	0	NA
Europe	11	4.0 (3.0, 5.0)	96.8	<0.001	0.076	0.876	4	3.1 (2.2, 4.1)
Asia	5	17.9 (6.6, 29.1)	96.8	<0.001	0.004	0.327	0	NA
Africa	1	33.3 (21.4, 45.2)	NA	NA	NA	NA	0	NA
Study period								
≤2010	4	2.0 (0.7, 3.3)	98.5	<0.001	0.701	0.718	0	NA
>2010	10	6.7 (5.0, 8.5)	89.5	<0.001	0.001	0.020	4	5.0 (3.2, 6.8)
Age								
2–12	7	2.7 (1.6, 3.8)	92.9	<0.001	0.005	0.015	3	1.8 (0.5, 3.1)
13–18	8	14.2 (9.3, 19.1)	90.4	<0.001	0.015	0.004	2	10.0 (4.9, 15.1)
ADHD diagnosis/criteria								
Standard diagnosis	15	5.9 (4.8, 7.0)	93.6	<0.001	0.001	0.023	6	4.5 (3.3, 5.7)
Standard criteria	10	3.3 (2.3, 4.4)	97.4	<0.001	0.111	0.472	3	2.7 (1.7, 3.8)
Standard diagnosis/criteria	8	4.0 (3.0, 5.0)	93.6	<0.001	0.090	0.266	3	3.4 (2.2, 4.6)
T1DM diagnosis/criteria								
Standard diagnosis	9	6.4 (4.8, 7.9)	94.8	<0.001	0.004	0.251	4	4.5 (2.9, 6.1)
Standard criteria	6	2.5 (1.5, 3.5)	97.3	<0.001	0.154	0.251	1	2.3 (1.2, 3.4)
Standard diagnosis/criteria	3	3.0 (2.0, 4.0)	94.6	<0.001	0.686	1.000	0	NA

Detection of publication bias by funnel plot, Begg test and Egger test (Supplementary Figures 3, 4). The fill prevalence of ADHD in the T1DM children and adolescent population obtained by the “trim and fill” method was 1.3%, whereas ASD had no fill prevalence in the T1DM adolescents.

Except ASD and ADHD, other NDDs retrieved in this study were not included in our meta-analysis.

### Type 1 diabetes mellitus in children and adolescents with neurodevelopmental disorders

There were two studies on T1DM among the included children and adolescents with ASD, which were conducted in the United States from 2001 to 2010. One was a clinical study and the other was a population-based study, with a prevalence rate of 0.93 and 0.67%, respectively. The quality evaluation was of general quality. There is only one study related to T1DM in children and adolescent ADHD patients, which is a population-based study conducted in China with a prevalence rate of 0.1%, and it is rated as good quality.

Except ASD and ADHD, there is no study on type 1 diabetes in children and adolescents with other neurodevelopmental disorders that met our inclusion criteria.

## Discussion

The results of the study showed that the prevalence of ASD and ADHD in T1DM children and adolescents was 1.1% (95% CI: 0.8–1.5) and 5.3% (95% CI: 4.3, 6.4), respectively, both higher than the global prevalence of ASD and ADHD in the general population in 2019 (0.4 and 1.1%), suggesting that T1DM has an impact on the prevalence of ASD and ADHD. Present study analyzed the prevalence of concurrent NDDs with T1DM in children and adolescents and explored the potential association between T1DM and NDDs.

**FIGURE 2:**
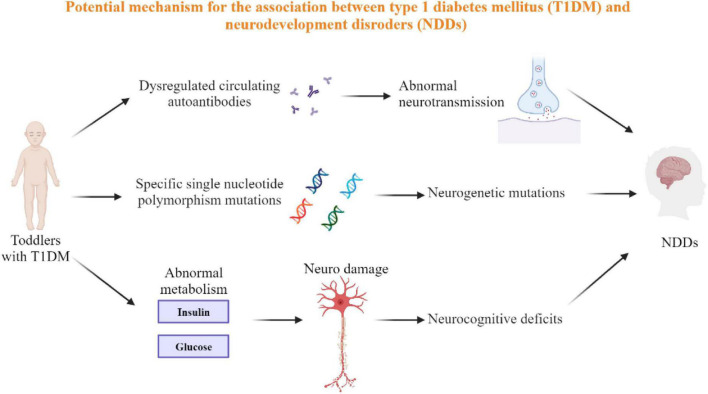
The potential mechanism of T1DM and NDDs. Three possible potential mechanisms for the interaction between type 1 diabetes and neurodevelopmental disorders are circulating autoantibody dysregulation, specific single nucleotide polymorphism mutations, and abnormal insulin and glucose concentrations, all of which involve substances that are associated with both T1DM and NDDs.

A growing number of studies have explored the relationship between metabolic disorders and psychiatric disorders, and there has been a gradual increase in research on the relationship between diabetes and psychiatric disorders, as well as a large body of literature exploring the effect of maternal diabetes on the development of neurodevelopmental disorders in offspring (61–65), but the detailed mechanisms remain unclear. Meanwhile, there are fewer original studies on the comorbid psychiatric disorders in patients with T1DM. Our systematic search strategy retrieves all available studies to comprehensively summarize the current evidence. Our subgroup analysis provides evidence for the increased prevalence of ADHD from childhood to age in patients with type 1 diabetes, but the relationship between ASD prevalence and age stratification needs to be further explored. Potential solid studies were identified through the “trim and fill” approach, which means there may be unpublished studies or less obvious results that were not included in our meta-analysis. However, the filled studies and their prevalence were not calculated by retrieval from existing data, and the “trim and fill” method used to adjust the mixed estimates was based on funnel plots (23), so the number of filled studies may not be true, and their estimates of prevalence may not be very accurate. Three mechanisms may be involved in the relationship between type 1 diabetes and neurodevelopmental disorders (Figure 2) (66, 67): Firstly, dysregulated circulating autoantibodies (GAD-Abs). Abnormalities of GAD65 and IA-2 antibodies (Abs) can be detected in the blood of patients with both type 1 diabetes and neurodevelopmental disorders. It was reported that GAD65 and IA-2 antibodies (Abs) are biomarkers for the development of type 1 diabetes mellitus (T1DM) in children and adults and can be detected in the blood months to years before the onset of symptoms (68). In a study with 20 children with ASD and 15 children with ADHD, immunoassays showed that 15% of children with ASD and 17% of children with ADHD had higher serum levels of GAD65 antibodies compared to controls (69). The neurotransmitter gamma amino butyric acid (GABA) is a major neurotropic agent in the central nervous system and plays a key role in the early development of the nervous system, while GAD65 is one of the two enzymes that catalyze the formation of GABA from glutamate (70); therefore, abnormalities in GAD65 may lead to abnormal development of the nervous system. Secondly, Specific single nucleotide polymorphism (SNP) mutations. Studies have shown that SNPs are significantly associated with a range of childhood and adult-onset psychiatric disorders (71), and copy number variants identified in genome-wide studies may contribute to the risk of multiple psychiatric disorders (72). At the same time, an increasing number of SNPs are significantly associated with the pathogenesis of diabetes (73). Thirdly, Abnormal insulin and glucose concentrations. Abnormal insulin and glucose concentrations include insulin deficiency, hyperglycemia, and medically induced hypoglycemia. There is now evidence that insulin plays an important role in neurodevelopment (74), and the effects of insulin overdose and resulting severe hypoglycemia on the central nervous system during T1DM treatment may lead to neurocognitive deficits (75), but there is no clear evidence to confirm the reliability of this theory. Similar to insulin, glucose metabolism is critical for brain development and function (35), and abnormal glucose in children with type 1 diabetes has a greater detrimental effect on central nervous system development (76). Also, a number of studies have shown that glucose abnormalities are associated with impaired neurocognitive function (77), which may lead to psychiatric disorders. In addition, Sillanpaa noted that poor social adjustment and reduced functioning in patients with T1DM may further increase their risk of developing major psychiatric disorders (78).

There may be some limitations to our analysis. First, the prevalence of mental developmental disorders among people with type 1 diabetes and the incidence of type 1 diabetes among people with mental developmental disorders may be underestimated, as there are still areas where low awareness of mental disorders or inadequate understanding of type 1 diabetes may lead to underdiagnosis and thus affect the accuracy of prevalence. Secondly, the literature is limited, and the results of our regional subgroup analysis for existing literature may not be applicable to all regions of the world. In addition, in the subgroup analysis results of prevalence of ASD in T1DM patients, the low prevalence in some age groups may lead to the unreliable prevalence of ASD in T1DM patients. However, mental disorders include six diseases, but there are few literatures related to some of them and more relevant researches are needed. Finally, recall bias can occur when recording situations through self-reports or parental reports.

In conclusion, this systematic review and meta-analysis suggests that type 1 diabetes is associated with the neurodevelopmental disorders in children and adolescents. There was a significant increase in the prevalence of ASD or ADHD in the adolescent T1DM population compared with that in the general population, and there was a trend for the prevalence of ASD and ADHD to increase with age in the T1DM population, but there is less literature on the association of the prevalence of T1DM in the children and adolescent neurodevelopmental disorders population. The potential link between the two disorders was also explored in this systematic review and meta-analysis, but the mechanisms behind T1DM and psychiatric disorders are not fully understood, and more high-quality studies are needed to elucidate the pathogenesis of T1DM and psychiatric disorders and to explore their intrinsic connections.

## Data availability statement

The original contributions presented in this study are included in the article/Supplementary material, further inquiries can be directed to the corresponding author/s.

## Author contributions

Y-ML and X-YL conceived the study design. C-YX and XL conducted data analysis. X-NX conducted study evaluation and wrote the manuscript. All authors have reviewed and approved the submitted version of the manuscript.
