# Supplementary material for: Association between type 1 diabetes and neurodevelopmental disorders in children and adolescents: A systematic review and meta-analysis
Source: Front Psychiatry. 2022 Nov 22;13:982696. doi: 10.3389/fpsyt.2022.982696 (PMC9722754; doi:10.3389/fpsyt.2022.982696)
Supplement: Supplementary file 1 [file Data_Sheet_1.docx]

**Supplementary Materials**

Table S1. Search strategy to identify studies reporting the neurodevelopmental disorders and T1DM.

Table S2. Characteristics of studies reporting neurodevelopmental disorders in T1DM population.

Table S3. Characteristics of studies reporting T1DM population in neurodevelopmental disorders population.

Table S4. Quality assessment for studies reporting ASD related articles

Table S5. Quality assessment for studies reporting ADHD related articles

Table S6. Quality assessment for studies reporting ID related articles

Figure S1. Pooled prevalence of ASD in T1DM population.

Figure S2. Pooled prevalence of ADHD in T1DM population.

Figure S3. Publication bias of studies on ASD in T1DM population. (A) Funnel plot; (B) Begg test; (C) Egger test.

Figure S4. Publication bias of studies on ADHD in T1DM population. (A) Funnel plot; (B) Begg test; (C) Egger test.

Table S1. Search strategy to identify studies reporting the relationship between neurodevelopmental disorders and T1DM.

|  | Headings* | Keywords |
| --- | --- | --- |
|  | 1.Adolescent [MeSH] | 2.Teenager [Title/Abstract]) OR adolescent [Title/Abstract] OR adolescence [Title/Abstract] OR teen [Title/Abstract] OR juvenile [Title/Abstract] OR child [Title/Abstract] OR children [Title/Abstract] OR youngster [Title/Abstract] OR youth [Title/Abstract] OR kid [Title/Abstract] OR kids [Title/Abstract] |
|  | 3.intellectual disability [MeSH] | 4. Intellectual Disabilities [Title/Abstract] OR Intellectual Development Disorder [Title/Abstract] OR Mental Retardation [Title/Abstract] OR Mental Deficiency [Title/Abstract] OR Mental Deficiencies [Title/Abstract] OR mental disability [Title/Abstract] OR ID [Title/Abstract] OR mental disabled [Title/Abstract] OR dysgnosia [Title/Abstract] OR dysnoesia [Title/Abstract] OR intellectual disability [Title/Abstract] OR intellectual impairment [Title/Abstract] |
|  | 5.communication disorder [Mesh] | 6. communication disorder [Title/Abstract] OR developmental communication disorder [Title/Abstract] OR communicative dysfunction [Title/Abstract] OR neurogenic communication disorder [Title/Abstract] OR communication disability [Title/Abstract] OR communication disabilities [Title/Abstract] OR communication barriers [Title/Abstract] OR communication impaired [Title/Abstract] OR impaired Verbal Communication [Title/Abstract] |
|  | 7.specific learning disorder [Mesh] | 8. specific learning disorder [Title/Abstract] OR learning disability [Title/Abstract] OR learning disorder [Title/Abstract] OR learning disabilities [Title/Abstract] OR specific learning difficulties [Title/Abstract] OR SPLD [Title/Abstract] OR LD [Title/Abstract] |
|  | 9. motor disorder [Mesh] | 10.dyskinesia [Title/Abstract] OR motor disturbance [Title/Abstract] OR dyskinesia [Title/Abstract] OR motor disorder [Title/Abstract] OR motor skills disorders [Title/Abstract] OR motor dysfunction [Title/Abstract] OR kinesipathy [Title/Abstract] OR dyskinetic [Title/Abstract] OR Movement Disorder [Title/Abstract] |
| ASD population | 11. Autism spectrum disorder [MeSH] | 12. Autism [title/abstract] OR Asperger syndrome [title/abstract] OR Asperger [title/abstract] OR ASD [title/abstract] OR autistic disorder [title/abstract] OR autism spectrum disorder [title/abstract] |
| ADHD population | 13. Attention deficit disorder with hyperactivity [Mesh] | 14. ADHD [title/abstract] OR attention deficit hyperactivity disorder [title/abstract] OR attention deficit disorder with hyperactivity [title/abstract] OR attention deficit disorder [title/abstract] OR hyperkinetic syndrome [title/abstract] OR syndrome hyperkinetic [title/abstract] OR overactive syndrome [title/abstract] OR attention deficit hyperkinetic disorder [title/abstract] OR hyperkinetic disorder [title/abstract] OR attention deficit disorder hyperactivity [title/abstract] OR attention deficit hyperactivity disorders [title/abstract] OR hyperactivity disorder [title/abstract] OR hyperactive syndrome [title/abstract] OR hyperkinetic syndromes [title/abstract] OR syndromes hyperkinetic [title/abstract] OR ADDH [title/abstract] OR adhd [title/abstract]. |
| Diabetes population | 15. Diabetes Mellitus [MeSH] | 16. diabetes [Title/Abstract] OR T1DM [Title/Abstract] OR DM [Title/Abstract] OR diabetic [Title/Abstract] OR glycuresis [Title/Abstract] |
| Combined Strategy | (1 OR 2) AND (3 OR 4) AND (15 OR16)  (1 OR 2) AND (5 OR 6) AND (15 OR16)  (1 OR 2) AND (7 OR 8) AND (15 OR16)  (1 OR 2) AND (9 OR 10) AND (15 OR16)  (1 OR 2) AND (11 OR 12) AND (15 OR16)  (1 OR 2) AND (13 OR 4) AND (15 OR16) | |

* Medical Subject Headings in PubMed as examples.

Table S2. Characteristics of studies reporting neurodevelopmental disorders in T1DM population.

| Authors and published years | Country | Study Setting | Data source | Study period | Sample size | Mean age (range) | Males % | NDDs diagnosis | NDDs criteria | Type of NDDs | Diabetes diagnosis | Diabetes criteria | Main outcome |
| --- | --- | --- | --- | --- | --- | --- | --- | --- | --- | --- | --- | --- | --- |
| Bethin et al. 2019 | USA | Population- based | The T1DX clinic network | 2016.06-2017.09 | 10,032 | 13 (<18 years old) | 52.0 | Questionnaire | DSM-5 | ASD | Diagnosed by doctors | Not specified | The prevalence of ASD in diabetes is 1.58% |
| Butwicka et al. 2015 | Sweden | Population- based | The SCDR, Swedish NDR, and the Swedish NPR | 1973-2009 | 17,122 | 9.3 years old | 54.1 | Not specified | ICD-9 code 299 and ICD-10 code F84 | ASD | Not specified | ICD-8: 250.00-250.09; ICD-9: 250A-250X; ICD-10: E10 | The prevalence of ASD in T1DM was 0.7% |
| Dybdal et al. 2018 | Denmark | Population- based | The Danish Civil Registration System and The NPR | 1996-2013 | 5,084 | 9.9 years old | 52.3 | Diagnosed by doctors | ICD-10: F84 | ASD | Diagnosed by doctors | ICD-10 codes E10-E16 | The prevalence of ASD in T1DM was 1.32% |
| Ferguson et al. 2019 | Scotland | Clinical-based | The Paediatric Diabetes Clinic in Highland region | Not specified | 124 | 5-16 years old | Not specified | Not specified | Not specified | ASD | Not specified | Not specified | The prevalence of ASD in diabetes is 12.1% |
| Freeman et al. 2005 | Canada | Clinical-based | The Diabetes Clinic at The Hospital for Sick Children in Toronto | 2002 | 984 | 4.8 (3.3-6.8) years old | Not specified | Diagnosed by either a psychiatrist or developmental pediatrician | Not specified | ASD | Diagnosed by either a psychiatrist or developmental pediatrician | Not specified | The prevalence of ASD in T1DM is 0.9% |
| Harjutsalo et al. 2006 | Finland | Population-based | The Prospective Childhood Diabetes Registry of Finland | Not specified | 5,178 | ≤14 years old | Not specified | Not specified | Not specified | ASD | Not specified | Not specified | The prevalence of ASD in T1DM was 0.14% |
| Liu et al. 2021 | Sweden | Population- based | Swedish health registers | 1973-2013 | 8,430 | 9.5 (Diagnosed <18) years old | 54.2 | Not specified | ICD-9: 299, ICD-10: F84 | ASD | Not specified | Not specified | The prevalence of ASD in T1DM was 1.0% |
| Liu et al. 2021 | Sweden | Population-based | Swedish registers | 1990-2013 | 11,326 | Diagnosed <18 years old | 55.4 | Not specified | ICD-9: 299A, 299.8;  ICD-10: F84.0, F84.1, F84.5, F84.8, F84.9 | ASD | Not specified | Not specified | The prevalence of ASD in T1DM was 0.79% |
| Stanek et al. 2016 | USA | Population- based | Not specified | Not specified | 2,610 | 18 mon-18 years old | Not specified | Not specified | Not specified | ASD | Not specified | Not specified | The prevalence of ASD in T1DM was 1.15% |
| Stanek et al. 2019 | USA | Clinical-based | The Barbara Davis Center for Diabetes | 2014.06-2015.06 | 2,597 | 12.4 (8 months-18 years) years old | 52.0 | Caregivers reported | ICD-9, DSM-IV | ASD | Not specified | Not specified | The prevalence of ASD in T1DM was 1.14% |
| Tojjar et al. 2019 | Sweden | Population- based | The national Better Diabetes Diagnosis database | 2005-2017 | 1,210 | Not specified | Not specified | Not specified | Not specified | ASD | Not specified | Not specified | The prevalence of ASD in T1DM was 2.98% |
| Bratina et al. 2015 | Slovenia | Population based | Available medical documentation | 2014-2015 | 101 | 11-17 years old | Not specified | Development and Well-Being Assessment (DAWBA) questionnaire and Psychiatric clinical examination | Not specified | ADHD | Development and Well-Being Assessment (DAWBA) questionnaire and Psychiatric clinical examination | Not specified | The prevalence of ADHD in T1DM is 11.88% |
| Butwicka et al. 2015 | Sweden | Population based | The Swedish Childhood Diabetes Register (SCDR), Swedish Natinal Diabetes Register (NDR), and the Swedish National Patient Register (NPR) | 1973-2009 | 17,122 | 9.3 years old | 54.1 | Not specified | ICD-9 code 314 and ICD-10 code F90 | ADHD | Not specified | ICD-8: 250.00-250.09; ICD-9: 250A-250X; ICD-10: E10 | The prevalence of ADHD in T1DM was 1.2% |
| Castillo et al. 2017 | Germany | Population based | A Nationawide Questionnaire Surveys | 2009-2010, 2012-2013 | 1,037 | 11-17 years old | 51.3 | Questionnaire | Not specified | ADHD | Questionnaire | Not specified | The prevalence of ADHD in T1DM is 6.6% |
| Duke et al. 2012 | USA | Population based | Not specified | Not specified | 64 | 15.1 years old | Not specified | Questionnaire | Not specified | ADHD | Not specified | Not specified | The prevalence of ADHD in T1DM was 28.4% |
| Dybdal et al. 2018 | Denmark | Population based | The Danish Civil Registration System and The National Patient Register (NPR) | 1996-2013 | 5,084 | 9.9 years old | 52.3 | Dignosed by doctors | ICD-10: F90 | ADHD | Dignosed by doctors | ICD-10 codes E10-E16 | The prevalence of ADHD in T1DM was 2.03% |
| Kapellen et al. 2016 | Germany | Clinical | IMS Disease Analyzer database | 2014.01-2014.12 | 3,668 | 0-18 years old | Not specified | Diagnosed by pediatrician | ICD-10 F90, -to F90.9 | ADHD | Diagnosed by pediatrician | ICD-10 E10. -to E10.91 | The prevalence of ADHD in T1DM was 4.2% |
| Kapellen et al. 2016 | Germany | Clinical | LRx database | 2014.01-2014.12 | 9,654 | 0-17 years old | Not specified | Diagnosed by pediatrician | ICD-10 F90, -to F90.9 | ADHD | Diagnosed by pediatrician | ICD-10 E10. -to E10.91 | The prevalence of ADHD in T1DM was 2.9% |
| Landau et al. 2019 | Israel | Population based | Not specified | Not specified | 111 | 13.09 (6-18) years old | Not specified | Not specified | Not specified | ADHD | Not specified | Not specified | The prevalence of ADHD in T1DM was 24% |
| Lin et al. 2019 | China | Population based | The Longitudinal Health Insurance Database for Illness Patients (LHID-CIP) | 1998-2011 | 2,852 | 10.9 years old | 46.2 | Not specified | ICD-9-CM code 314 | ADHD | Not specified | ICD-9-CM codes 250.x1 and 250.x3 | The prevalence of ADHD in T1DM was 1.05% |
| Lindblad et al. 2017 | Sweden | Population based | A previous study in two counties in Sweden | 2013-2014 | 175 | 5-16 years old | Not specified | The Wschsler Intelligence Scale for Children - Four Edition, parents reported | Not specified | ADHD | Not specified | Not specified | The prevalence of ADHD in T1DM was 4.57% |
| Liu et al. 2021 | Sweden | Population based | Swedish health registers | 1973-2013 | 8,430 | 9.5 (Dignosed <18) years old | 54.2 | Not specified | ICD-9: 314; ICD-10: F90 | ADHD | Not specified | Not specified | The prevalence of ADHD in T1DM was 3.52% |
| Liu,S et al. 2021 | Sweden | Population based | Swedish registers | 1973-2013 | 11,326 | Dignosed <18 years old | 55.4 | Taking ADHD medication | ICD-9: 314; ICD-10: F90 | ADHD | Not specified | Not specified | The prevalence of ADHD in T1DM was 3.66% |
| Macek et al. 2019 | Slovenia | Population based | The department of endocrinology, diabetes and metabolic diasases at University Children's Hospital Ljubljana (UCHL) | 2013.09-2014.08 | 101 | 11-17 years old | 56.4 | Development and Well-Being Assessment (DAWBA) questionnaire and psychiatric clinical examination | DSM-IV | ADHD | Development and Well-Being Assessment (DAWBA) questionnaire and Psychiatric clinical examination | Not specified | The prevalence of ADHD in T1DM was 11.88% |
| Mazor et al. 2019 | Israel | Clinical | 3 paediatric diabetes clinics | Not specified | 111 | 14.6 (6-18) years old | Not specified | ADHD screening questionnaire | Not specified | ADHD | Modified Diabetes QOL (DQOL) questionnaire | Not specified | The prevalence of ADHD in T1DM was 24.32% |
| Mazor et al. 2021 | Israel | Clinical | The paediatric diabetes teams from 3 multidisciplinary clinics | Not specified | 121 | 13.08 (6-18) years old | 57.0 | DSM5 screening questionnaire for ADHD | Not specified | ADHD | Modified Diabetes QOL (DQOL) questionnaire | Not specified | The prevalence of ADHD in T1DM was 32.23% |
| Merzon et al. 2017 | Israel | Population based | The Leumit Health Service | Not specified | 230 | 12.9 (< 18) years old | 49.9 | Not specified | Not specified | ADHD | Not specified | Not specified | The prevalence of ADHD in T1DM was 10.5% |
| Nylander et al. 2017 | Sweden | Clinical-based | 4 diabetes centers in Sweden | 2013.03-2015.02 | 241 | 15.2 (12-18) years old | 49.4 | ADHD-RS, BRIEF-SR, self-report, parent-report | DSM-IV | ADHD | Not specified | Not specified | The prevalence of ADHD in T1DM was 5.39% |
| Nylander et al. 2018 | Sweden | Clinical-based | The 3 Departments of Pediatrics in Southern Stockholm; the Astrid Lindgren Children’s Hospital in Huddinge and the Sachs Children and Youth Hospital at the South Hospital and the Pediatric Clinic at Uppsala University Children’s Hospital in Uppsala, Sweden | 2013.03-2015.02 | 166 | 15.6 (12-18) years old | 53.8 | ADHD RS-IV by parents and the BRIEF-SR | Not specified | ADHD | Not specified | Not specified | The prevalence of ADHD in T1DM was 5.42% |
| Sakhr et al. 2020 | Egypt | Clinical-based | The outpatient paediatric endocrinology and neuropsychiatric clinics at Qena University Hospital, South Valley University | 2018.02-2019.03 | 60 | 10.29 (6-18) years old | 50 | An interview with two specialized expert doctors | ADHD-SC4, DSM-IV | ADHD | Not specified | The 2012 criteria of the American Diabetes Association | The prevalence of ADHD in T1DM was 33.33% |
| Vinker et al. 2019 | Israel | Population- based | The LHS database | 2016.01-2016.12 | 230 | 12.96 (5- 18) years old | 49.1 | Diagnosis made by paediatric psychiatrists, neurologists and paediatricians or family medicine specialist with recognized ADHD/ neurodevelopmental training and purchased at least 3 prescriptions of ADHD medication. | Not specified | ADHD | Dignosed by community family physicians, pediatricians, endocrinologists, or psychiatrists | Not specified | The prevalence of ADHD in T1DM was 10.4% |
| Vinker-Shuster et al. 2022 | Israel | Population- based | The LHS database | 2018 | 789 | Not specified | Not specified | Not specified | Not specified | ADHD | Not specified | Not specified | The prevalence of ADHD in T1DM was 9.5% |
| Yazar et al. 2019 | Turkey | Clinical-based | The Pediatric Endocrinology and Metabolism Clinic of Necmettin Erbakan University hospital | 2017.02-2017.08 | 61 | 12.4 (7-17) years old | 47.0 | Semi-structured psychiatric interview using Turkish version of Schedule for Affective Disorders and Schizophrenia for School-Age Children (Kiddie-SADS Lifetime Version). | DSM-3, DSM-4, DSM-V | ADHD | Diagnosed by doctors | Not specified | The prevalence of ADHD in T1DM was 24.59% |
| Butwicka et al. 2015 | Sweden | Population- based | The SCDR, Swedish NDR, and the Swedish NPR | 1973-2009 | 17,122 | 9.3 years old | 54.1 | Not specified | ICD-8 codes 310-315, ICD-9 codes 317-319, and ICD-10 codes F70-F79 | ID | Not specified | ICD-8: 250.00-250.09; ICD-9: 250A-250X; ICD-10: E10 | The prevalence of ID in T1DM was 0.7% |
| Dybdal et al. 2018 | Denmark | Population- based | The Danish Civil Registration System and The NPR | 1996-2013 | 5,084 | 9.9 years old | 52.3 | Diagnosed by doctors | ICD-10: F70-F79 | ID | Diagnosed by doctors | ICD-10 codes E10-E16 | The prevalence of ID in T1DM was 0.47% |
| Liu et al. 2021 | Sweden | Population- based | Swedish health registers | 1973-2013 | 8,430 | 9.5 (Diagnosed <18) years old | 54.2 | Not specified | ICD-9: 299, ICD-10: F84 | ID | Not specified | Not specified | The prevalence of ASD in T1DM was 7.76% |
| Liu,S et al. 2021 | Sweden | Population- based | Swedish registers | 1990-2013 | 11,326 | Diagnosed <18 years old | 55.4 | Not specified | ICD-8 codes 310-315, ICD-9 codes 317-319, and ICD-10 codes F70-F79 | ID | Not specified | Not specified | The prevalence of ID in T1DM was 0.47% |

Abbreviations: ASD, Autism Spectrum Disorder; T1DM, Type 1 Diabetes Mellitus; NDDs, Neurodevelopmental disorders; T1DX, T1D Exchange Clinic Network; DSM, Diagnostic and Statistical Manual; ICD, International Classification of Diseases; BRIEF-SR, Behavior Rating Inventory of Executive Function Self-report version; ADHD-RS-IV, ADHD Rating Scale-IV; ADHD-SC4, ADHD Symptom Checklist-4; LHS, Leumit-Health-Services; SADS, Schedule for Affective Disorders; SCDR, Swedish Childhood Diabetes Register; NDR, National Diabetes Register ;NPR, National Patient Register.

Table S3. Characteristics of studies reporting T1DM population in neurodevelopmental disorders population.

| Authors and published years | Country | Setting | Data source | Study period | Sample size | Mean age (range) | Males % | NDDs diagnosis | NDDs criteria | Type of NDDs | Diabetes diagnosis | Diabetes criteria | Main outcome |
| --- | --- | --- | --- | --- | --- | --- | --- | --- | --- | --- | --- | --- | --- |
| Kohane et al. 2012 | USA | Population based | The SHRINE System | 2001-2010 | Not specified | 0-17 years old | Not specified | Not specified | ICD-9 | ASD | Not specified | ICD-9 | The prevalence of T1DM in ASD is 0.67% |
| Supekar et al. 2017 | USA | Clinical | One of the two Stanford University Medical Center Hospitals: Lucile Packard Children’s and Stanford Hospital and Clinics | Not specified | Not specified | 0-18 years old | Not specified | Not specified | ICD-9 | ASD | Not specified | Not specified | The prevalence of T1DM in ASD is 0.93% |
| Chen et al. 2013 | China | Population based | The LHID2005 | Not specified | 4,302 | 8.56 (5-15) years old | 80.0 | Not specified | ICD-9-CM code 314.00 or 314.01 | ADHD | Not specified | ICD-9-CM code 250 | The prevalence of T1DM in ADHD is 0.1% |

Abbreviations: T1DM, Type 1 Diabetes Mellitus; ASD, Autism Spectrum Disorder; ADHD, Attention deficit hyperactivity disorder; NDDs, Neurodevelopmental disorders; ICD, International Classification of Diseases; SHRINE, Shared Health Research Informatics Network; LHID, Longitudinal Health Insurance Database 2005.

Table S4. Quality assessment for studies reporting ASD related articles

|  | Q1 Research question | Q2 Study population | Q3 Participation | Q4.1 Sampling | Q4.2  Inclusion and exclusion criteria | Q5 Sample size | Q6 Exposure | Q7 Timeframe | Q8 Exposure level | Q9 Exposure Measures | Q10 Assessment Validation | Q11 Outcome measures | Q12 Blindness | Q13 Follow-up | Q14 Confounding variables | Rating |
| --- | --- | --- | --- | --- | --- | --- | --- | --- | --- | --- | --- | --- | --- | --- | --- | --- |
| Bethin et al. 2019 | 1 | 1 | 1 | 1 | 1 | 0 | 1 | 1 | NA | 1 | 0 | 1 | NA | NA | 1 | 10 |
| Butwicka et al. 2015 | 1 | 1 | 1 | 1 | NR | 0 | 1 | 1 | NA | 1 | 0 | 1 | NA | NA | 1 | 9 |
| Dybdal et al. 2018 | 1 | 1 | 1 | 1 | NR | 0 | 1 | 1 | NA | 1 | 0 | 1 | NA | NA | 1 | 9 |
| Freeman et al. 2005 | 0 | 1 | 1 | 1 | NR | 0 | 1 | 1 | NA | 1 | 0 | 1 | NA | NA | 0 | 7 |
| Liu et al. 2021 | 1 | 1 | 1 | 1 | NR | 0 | 1 | 1 | NA | 1 | 1 | 1 | NA | 0 | 1 | 10 |
| Liu,S et al. 2021 | 1 | 1 | 1 | 1 | 1 | 0 | 1 | 1 | NA | 1 | 0 | 1 | NA | NA | 1 | 10 |
| Stanek et al. 2019 | 1 | 1 | 1 | 1 | 1 | 0 | 1 | 1 | NA | 0 | 0 | 1 | NA | NA | 0 | 8 |
| Kohane et al. 2012 | 1 | 1 | 1 | 1 | 0 | 0 | 1 | 1 | NA | 1 | 0 | 1 | NA | NA | 0 | 8 |
| Supekar et al. 2017 | 0 | 1 | 1 | 0 | 1 | 0 | 1 | 1 | NA | 1 | 0 | 0 | NA | NA | 0 | 6 |

Abbreviations: NA, Not Applicable.

Table S5. Quality assessment for studies reporting ADHD related articles

|  | Q1 Research question | Q2 Study population | Q3 Participation | Q4.1 Sampling | Q4.2  Inclusion and exclusion criteria | Q5 Sample size | Q6 Exposure | Q7 Timeframe | Q8 Exposure level | Q9 Exposure Measures | Q10 Assessment Validation | Q11 Outcome measures | Q12 Blindness | Q13 Follow-up | Q14 Confounding variables | Rating |
| --- | --- | --- | --- | --- | --- | --- | --- | --- | --- | --- | --- | --- | --- | --- | --- | --- |
| Butwicka et al. 2015 | 1 | 1 | 1 | 1 | NR | 0 | 1 | 1 | NA | 1 | 0 | 1 | NA | NA | 1 | 9 |
| Dybdal et al. 2018 | 1 | 1 | 1 | 1 | NR | 0 | 1 | 1 | NA | 1 | 0 | 1 | NA | NA | 1 | 9 |
| Kapellen et al. 2016 | 1 | 1 | 0 | 1 | NR | 0 | 1 | 1 | NA | 1 | 0 | 1 | NA | NA | 0 | 7 |
| Kapellen et al. 2016 | 1 | 1 | 0 | 1 | NR | 0 | 1 | 1 | NA | 1 | 0 | 1 | NA | NA | 0 | 7 |
| Lin et al. 2019 | 1 | 1 | 1 | 1 | 1 | 0 | 1 | 1 | NA | 1 | 0 | 1 | NA | 0 | 1 | 10 |
| Lindblad et al. 2017 | 1 | 1 | 0 | 1 | NR | 0 | 1 | 1 | NA | 1 | 0 | 1 | NA | NA | 0 | 7 |
| Liu et al. 2021 | 1 | 1 | 1 | 1 | NR | 0 | 1 | 1 | NA | 1 | 1 | 1 | NA | 0 | 1 | 10 |
| Liu,S et al. 2021 | 1 | 1 | 1 | 1 | 1 | 0 | 1 | 1 | NA | 1 | 0 | 1 | NA | NA | 1 | 10 |
| Macek et al. 2019 | 1 | 1 | 1 | 1 | 1 | 0 | 1 | 1 | NA | 1 | 0 | 1 | NA | NA | 0 | 9 |
| Mazor et al. 2021 | 1 | 1 | 1 | 1 | 1 | 0 | 1 | 1 | NA | 1 | 0 | 1 | NA | NA | 1 | 10 |
| Nylander et al. 2017 | 1 | 1 | 1 | 1 | NR | 0 | 1 | 1 | NA | 1 | 0 | 1 | NA | NA | 1 | 9 |
| Nylander et al. 2018 | 1 | 1 | 1 | 1 | 1 | 0 | 1 | 1 | NA | 1 | 0 | 1 | NA | NA | 1 | 10 |
| Sakhr et al. 2020 | 1 | 1 | 1 | 1 | NR | 0 | 1 | 1 | NA | 1 | 0 | 1 | NA | NA | 0 | 8 |
| Vinker et al. 2019 | 1 | 1 | 1 | 1 | NR | 0 | 1 | 1 | NA | 1 | 0 | 1 | NA | NA | 1 | 9 |
| Yazar et al. 2019 | 1 | 1 | 1 | 1 | 1 | 0 | 1 | 1 | NA | 1 | 0 | 1 | NA | NA | 0 | 9 |
| Chen et al. 2013 | 1 | 1 | 1 | 1 | 1 | 0 | 1 | 1 | NA | 1 | 0 | 1 | NA | NA | 1 | 10 |

Abbreviations: NA, Not Applicable.

Table S6. Quality assessment for studies reporting ID related articles

|  | Q1 Research question | Q2 Study population | Q3 Participation | Q4.1 Sampling | Q4.2  Inclusion and exclusion criteria | Q5 Sample size | Q6 Exposure | Q7 Timeframe | Q8 Exposure level | Q9 Exposure Measures | Q10 Assessment Validation | Q11 Outcome measures | Q12 Blindness | Q13 Follow-up | Q14 Confounding variables | Rating |
| --- | --- | --- | --- | --- | --- | --- | --- | --- | --- | --- | --- | --- | --- | --- | --- | --- |
| Butwicka et al. 2015 | 1 | 1 | 1 | 1 | NR | 0 | 1 | 1 | NA | 1 | 0 | 1 | NA | NA | 1 | 9 |
| Liu et al. 2021 | 1 | 1 | 1 | 1 | NR | 0 | 1 | 1 | NA | 1 | 1 | 1 | NA | 0 | 1 | 10 |
| Liu,S et al. 2021 | 1 | 1 | 1 | 1 | 1 | 0 | 1 | 1 | NA | 1 | 0 | 1 | NA | NA | 1 | 10 |
| Dybdal et al. 2018 | 1 | 1 | 1 | 1 | NR | 0 | 1 | 1 | NA | 1 | 0 | 1 | NA | NA | 1 | 9 |

Abbreviations: NA, Not Applicable.


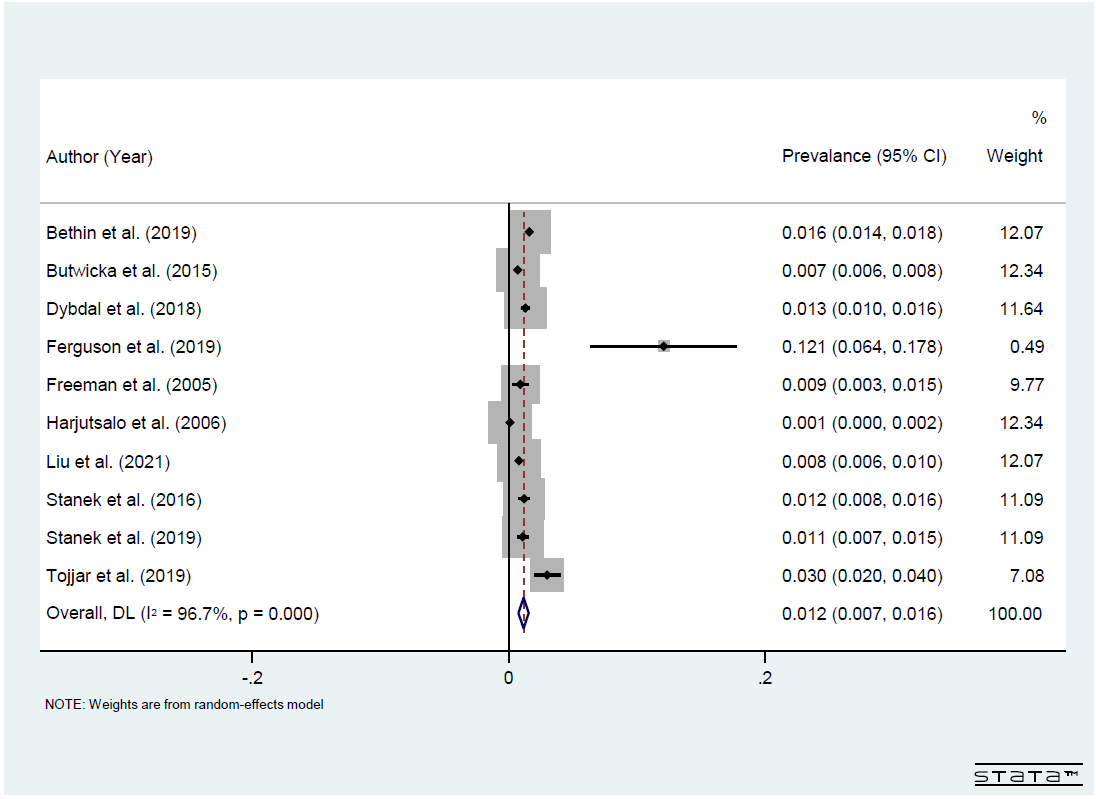


Figure S1. Forest plot of the pooled prevalence of ASD in T1DM population.


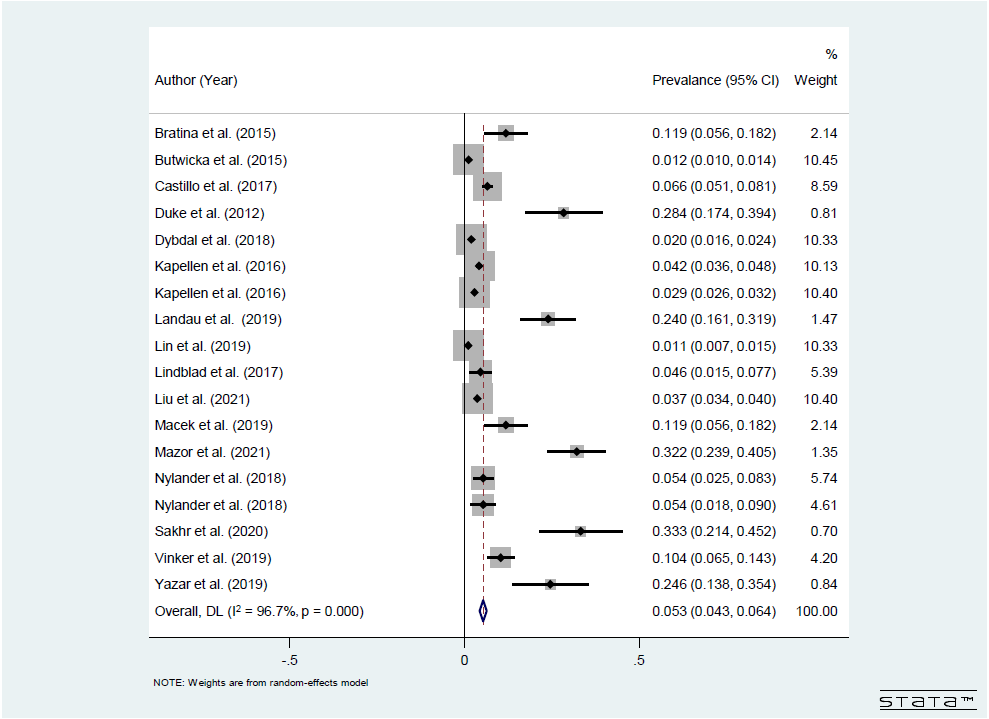


Figure S2. Pooled prevalence of ADHD in T1DM population.

Figure S3. Publication bias of studies on ASD in T1DM population. (A) Funnel plot; (B) Begg test; (C) Egger test.

Figure S4. Publication bias of studies on ADHD in T1DM population. (A) Funnel plot; (B) Begg test; (C) Egger test.
